# Non-Antibiotic Compounds Synergistically Kill Chronic Wound-Associated Bacteria and Disrupt Their Biofilms

**DOI:** 10.3390/pharmaceutics15061633

**Published:** 2023-05-31

**Authors:** Lucy Coleman, James R. G. Adams, Will Buchanan, Tao Chen, Roberto M. La Ragione, Lian X. Liu

**Affiliations:** 1 School of Chemistry & Chemical Engineering, Faculty of Engineering and Physical Science, University of Surrey, Guildford GU2 7XH, UK; t.chen@surrey.ac.uk; 2 School of Veterinary Medicine, Faculty of Health and Medical Sciences, University of Surrey, Guildford GU2 7AL, UK; ja01329@surrey.ac.uk (J.R.G.A.); r.laragione@surrey.ac.uk (R.M.L.R.); 3 Avian Immunology, The Pirbright Institute, Woking GU24 0NE, UK; 4 Phytoceutical Ltd., Midhurst, West Sussex GU29 9DJ, UK; will@phytoceutical.co.uk; 5 School of Biosciences, Faculty of Health and Medical Sciences, University of Surrey, Guildford GU2 7XH, UK

**Keywords:** non-antibiotics, antibacterial, antibiofilm, PHMB, TPGS, retinol, curcumin, Minimum Inhibitory Concentration (MIC)

## Abstract

Chronic wounds and their treatment present a significant burden to patients and healthcare systems alike, with their management further complicated by bacterial infection. Historically, antibiotics have been deployed to prevent and treat infections, but the emergence of bacterial antimicrobial resistance and the frequent development of biofilms within the wound area necessitates the identification of novel treatment strategies for use within infected chronic wounds. Here, several non-antibiotic compounds, polyhexamethylene biguanide (PHMB), curcumin, retinol, polysorbate 40, ethanol, and D-α-tocopheryl polyethylene glycol succinate 1000 (TPGS) were screened for their antibacterial and antibiofilm capabilities. The minimum inhibitory concentration (MIC) and crystal violet (CV) biofilm clearance against two bacteria frequently associated with infected chronic wounds, *Staphylococcus aureus* and *Pseudomonas aeruginosa*, were determined. PHMB was observed to have highly effective antibacterial activity against both bacteria, but its ability to disperse biofilms at MIC levels was variable. Meanwhile, TPGS had limited inhibitory activity but demonstrated potent antibiofilm properties. The subsequent combination of these two compounds in a formulation resulted in a synergistic enhancement of their capability to kill both *S. aureus* and *P. aeruginosa* and disperse their biofilms. Collectively, this work highlights the utility of combinatory approaches to the treatment of infected chronic wounds where bacterial colonization and biofilm formation remains significant issues.

## 1. Introduction

Chronic wounds are wounds that do not heal in the expected timeframe (usually defined as between 4 weeks and 3 months), resulting in a lack of sustainable anatomical or functional tissue [1]. The development of chronic wounds leads to a significant burden on the individual, and ineffective wound care can lead to amputation, loss of mobility, and serious psychological ramifications stemming from the impact of the condition on the individual’s lifestyle [2,3]. Chronic wounds and their treatment reportedly cost healthcare systems USD 28 billion and USD 31.7 billion for primary and secondary diagnoses, respectively [2]. However, as elderly populations are at higher risk of developing chronic wounds, and the proportion of people aged 65 and over is predicted to increase from 1 in 11 in 2019 to 1 in 6 in 2050, this cost will likely increase dramatically without the development of novel interventions [2,4].

Effective wound healing is complicated by the presence of bacteria within the wound, causing inflammation and tissue damage. Chronic wounds are highly susceptible to infection due to the loss of the innate protective barrier, allowing the entry of pathogenic microorganisms into the sterile subdermal space [5]. Of the bacteria associated with chronic wound infections, the opportunistic pathogens *Staphylococcus aureus* and *Pseudomonas aeruginosa* are the most common [6]. Both Gram-positive *S. aureus* and Gram-negative *P. aeruginosa* are also associated with biofilm production and the recent surge in the emergence of antimicrobial and multidrug-resistant strains [5]. Biofilms consist of a range of bacterial and microbial communities connected by a robust layer of extracellular polymeric substances (EPS): primarily polysaccharides, proteins, nucleic acids, and lipids [7]. The formation of biofilms is seen as a key factor in the disruption of chronic wound healing, with bacterial biofilm structures presenting within 6% of acute wounds but 90% of chronic ones [8]. The biofilm community adheres to the wound surface, sequestering nutrients and resources from the host which would otherwise contribute to wound healing [8]. Microbes can form biofilms on biotic or abiotic surfaces even under adverse conditions, such as high salinity or extreme pH, with the process generally observed to consist of several distinct stages [9]. This includes the attachment of planktonic or aggregated microbes through adhesins on the cell surface. This is then followed by growth and accumulation, wherein the bacterial community expands through replication and recruitment of nearby microbial cells [10]. Following expansion, the biofilm reaches maturity, and the microbial community undergoes disaggregation and detachment, leaving the biofilm as bacterial aggregates or planktonic cells [10,11]. Detached single and aggregated cells may then migrate, assisted by the expression of translocation-associated genes, allowing the colonization of new niches [11]. Biofilm communities are generally distinct, with characteristics dependent on the microbial composition, but consist of 90% EPS and are frequently associated with greatly increased tolerance and resistance of antibiotics and biocides [11,12,13]. This reduced susceptibility to antimicrobials is partly due to the biofilm limiting diffusion of compounds deep within the microbial community. Moreover, the hypoxic and acidic conditions within the biofilm microenvironment promote the expression of antimicrobial-inactivating hydrolases [11]. The intrinsic resilience of biofilms contributes to the difficulties faced during the treatment of chronically infected wounds.

A wide range of treatments are employed in the management of infected chronic wounds, dependent on the nature of the injury and the microbial community/biofilm present [14]. Antibiotics can be employed as a standalone treatment [15] or alongside debridement, a treatment that involves thoroughly cleaning the wound and the chemical or mechanical removal of all unviable, infected, or thickened tissues from the wound area [8,16]. However, up to 50% of antibiotic treatments may be unnecessary, resulting in the potential development of antimicrobial resistance [17]. Furthermore, mature biofilms have increased antimicrobial resistance [16]. Alternatively, antiseptics are employed due to their broad spectrum effectiveness against bacterial pathogens and reduced likelihood of the development of resistance [18]. Indeed, the antiseptic polyhexamethylene biguanide (PHMB) has found frequent and historic use within domestic, industrial, and medical processes as a biocide, yet bacterial resistance has not been reported [19,20,21]. PHMB has been routinely employed in the treatment of clinically infected chronic wounds as an antimicrobial wash and has been demonstrated to have a significant bactericidal effect on *P. aeruginosa* and *S. aureus* in an ex vivo porcine explant mature wound model [22] and in vitro bovine mammary epithelial (Mac-T) model [23], respectively. However, in both studies, PHMB reduced but did not clear the mature biofilm completely [22,23]. This is of concern as it may allow the reconstitution of the biofilm and microbial community by the remaining or other bacterial populations [8].

Due to the difficulties associated with the clearance of biofilms, there has been increased interest in the use of novel compounds with the capacity to modify biofilm permeability in combination with an antimicrobial. These formulations aim to eliminate microbes present within the biofilm community by a dual-action approach where one compound facilitates the penetration of the other to enhance activity and disperse biofilms [24]. Surfactants such as polysorbate 40 have been demonstrated to inhibit *P. aeruginosa* biofilm formation [25], while the combination of the surfactant poloxamer 407 with the antiseptic chlorhexidine resulted in the eradication of both *Escherichia coli* and *P. aeruginosa* biofilms [26]. Meanwhile, surfactants such as D-α-Tocopherol polyethylene glycol 1000 succinate (TPGS) have also been suggested for use within wound healing due to their properties as a permeation enhancer and solubilizing agent in drug delivery [27,28], particularly as micelles [29].

Naturally derived compounds, such as retinol, present in animal products and as chemical precursors in fruits and vegetables, have also been identified as potential candidate compounds for inclusion in chronic wound treatments. This is due to retinol’s antibacterial effect against various Gram-positive and Gram-negative bacteria, including both *S. aureus* and *P. aeruginosa* [30]. In addition, curcumin, a lipophilic bioactive compound obtained from the rhizome of *Curcuma longa* Linnean plant [31], has also displayed both antiseptic and potential antibiofilm abilities [32], being tested against multiple species of bacteria and fungi [33,34].

In this study, we have investigated the antibacterial and antibiofilm properties of PHMB, curcumin, retinol, and polysorbate 40, ethanol, and D-α-tocopheryl polyethylene glycol succinate 1000 (TPGS) individually and/or in combination against *S. aureus* and *P. aeruginosa* and their biofilms. This research aimed to inform the development of antibiotic-free alternatives for the elimination of bacteria and inhibition/removal of their associated biofilms.

## 2. Materials and Methods

### 2.1. Chemicals

**Figure 1:**
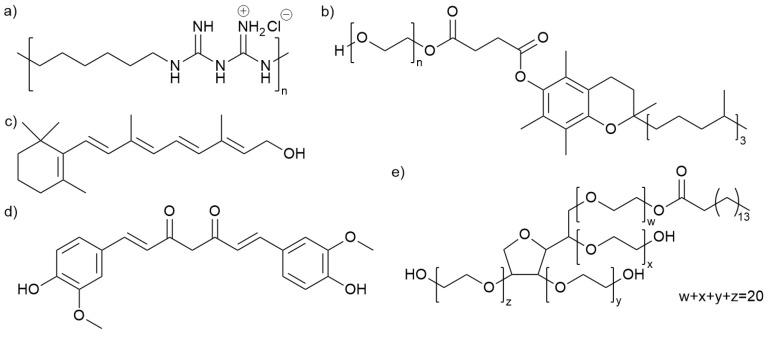
Skeletal structures of candidate compounds: (**a**) Polyhexamethylene biguanide (PHMB); (**b**) D-α-Tocopherol polyethylene glycol 1000 succinate (TPGS); (**c**) retinol; (**d**) curcumin; (**e**) Polysorbate 40.

Polyhexamethylene biguanide (PHMB) was purchased from Lonza, UK. Retinol, curcumin, D-α-Tocopherol polyethylene glycol 1000 succinate (TPGS), polysorbate 40, and ethanol were purchased from Sigma-Aldrich (Gillingham, UK). Table 1 shows the initial ‘stock’ solutions of the compounds with the corresponding solvents and concentrations. Figure 1 shows the structure of the candidate compounds. With TPGS, two different types of stock solutions were prepared; one was obtained by simple mixing of TPGS with Milli-Q water, and the other was prepared using high-speed (5000 rpm) homogenization, enabling the formation of TPGS micelles (TPGS-M).

### 2.2. Bacterial Isolates and Growth Conditions

*S. aureus* (NCTC12493) and *P. aeruginosa* (NCTC12903) were stored in Pro-Lab Diagnostics Microbank (Fisher, Basingstoke, UK) tubes at −80 °C before streaking onto Nutrient Agar (Oxoid, Basingstoke, UK) and incubated at 37 °C, aerobically for 18 h.

### 2.3. Minimum Inhibitory Concentration (MIC)

Minimum inhibitory concentrations (MICs) were determined using the broth microdilution method [35]. Briefly, 10 mL of Mueller–Hinton broth (MHB) (Oxoid, Basingstoke, UK) in a 50 mL centrifuge tube was inoculated with a single bacterial colony using a sterile loop, and incubated for 18 h, shaking at 200 RPM at 37 °C. The bacterial suspension was then adjusted through the addition of fresh LB media until a turbidity equivalent to that of a 0.5 McFarland Standard was reached, and 100 µL of this bacterial suspension was added to a 96-well microtiter plate containing 100 µL of test compounds (Table 1) which had been diluted two-fold in MHB to generate a range of concentrations or to triplicate wells containing 100 µL MHB media alone to act as a positive growth control. Additionally, media without bacterial inoculation was added to determine sterility and act as a negative control. Plates were incubated aerobically for 18 h at 37 °C. Following incubation, MIC was determined through visual observation of no turbidity within the media in the well and confirmed using a TECAN plate reader at OD600 nm, where inhibition was characterized as OD600 < 50% of the triplicate positive growth control wells consisting of MHB media inoculated with the bacterial suspension.

### 2.4. Minimum Bactericidal Concentration (MBC)

After performing the MIC assay as above, each well was homogenized using a pipette, and the Miles and Misra method [36] was used to determine the MBC. Briefly, 20 µL from each well was transferred to Mueller–Hinton agar (MHA) plates and incubated aerobically for a further 18 h. The lowest concentration of compound to result in no bacterial growth was determined as the MBC.

### 2.5. Bacterial Kill Curves

A single bacterial colony was transferred into 10 mL Luria–Bertani (LB) broth (Oxoid, Basingstoke, UK) within a 50 mL centrifuge tube and incubated aerobically, shaking at 200 RPM at 37 °C for 18 h. To a 1.5 mL microcentrifuge tube, 100 µL of the 18 h culture was transferred and centrifuged at 13,300× *g* for 5 min at room temperature. Before the supernatant was removed. The bacterial pellet was resuspended in 1 mL PBS (1:100 dilution), and 20 μL of bacterial suspension was added to 180 μL LB broth and Dulbecco′s Modified Eagle′s Medium/F-12, GlutaMAX™ supplement (DMEM) (Fisher, Basingstoke, UK) for *P. aeruginosa* and *S. aureus*, respectively. The 96-well plate was incubated statically at 37 °C under aerobic conditions. Each hour, the 200 µL samples were removed from the 96-well plate, transferred to a 1.5 mL microcentrifuge tube, and stored at 4 °C. At three hours post-inoculation (mid-log phase), 1 μL of 20% PHMB was added to each well, and sampling continued for an additional three hours. Once all samples were collected, viable counts were determined using the Miles and Misra method [36].

### 2.6. Crystal Violet Biofilm Clearance Assays

**Figure 2:**
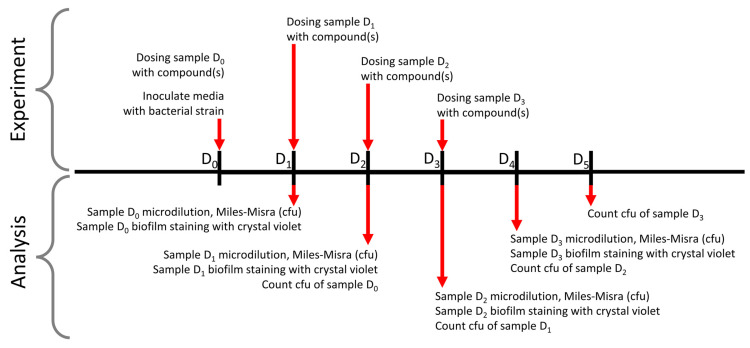
Timeline of biofilm growth experiments and concurrent crystal violet assay analysis performed. Symbols are as follows: D_n_, n days after inoculation of bacteria.

This methodology was adapted from that described by Merritt et al., 2005 [37]. Briefly, biofilms were generated using a single bacterial colony to inoculate 10 mL LB broth within a 50 mL centrifuge tube and incubated aerobically, shaking at 200 RPM at 37 °C for 18 h. Following this, 100 µL of overnight culture was transferred to a 1.5 mL microcentrifuge tube and spun at 13,300× *g* for 5 min at room temperature before the supernatant was removed, and the bacterial pellet was washed twice in 1 mL PBS. The washed pellet was then reconstituted in 100 µL PBS which was added to either 9.9 mL (1:100) DMEM or M9 minimal media +1% glucose for *P. aeruginosa* and *S. aureus*, respectively. At D0, 100 µL of inoculated media was added to each well of a 96-well microtiter plate. Candidate compounds (Table 1) were added to experimental wells (D0). Plates were incubated at 37 °C aerobically for 24 h before biofilm formation was determined within control and experimental wells on D1 by removing planktonic bacteria via pipetting and then washing the biofilms with 100 µL Milli-Q water. To each well, 125 µL of 0.1% crystal violet (CV) solution is then added and incubated at room temperature for 10 min. The 0.1% CV solution was removed, and the well was washed with water until the waste liquid ran clear. The stain was left to air-dry before 200 µL 30% acetic acid was added to each well, followed by incubation for 15 min on a plate shaker (room temperature, 200 rpm). Following solubilization, 125 µL of each sample was transferred to a new 96-well plate, and the OD was read at 600 nm. This was repeated for D1 and D2.. Figure 2 illustrates the timeline of the experiment and subsequent analysis.

### 2.7. Biofilm Bacterial Survival Assays

Following the generation of biofilms, as previously described in Section 2.6, bacterial biofilms were disrupted and homogenized by vigorous pipetting at the time of sampling. The bacterial suspension was then diluted in sterile PBS, following which viable bacterial counts were determined using the Miles and Misra method [33].

## 3. Results

### 3.1. Antibacterial Activity of Individual Compounds

The six compounds listed in Table 1 were screened using the broth microdilution method for their antimicrobial activity against two bacterial strains frequently associated with clinical infection within chronic wounds [6,38], *S. aureus* (NCTC12493) and *P. aeruginosa* (NCTC12903). Curcumin diluted in both water and ethanol was investigated, but only TPGS and not TPGS-M were assayed as the arrangement of TPGS is unlikely to impact antimicrobial activity. Of the candidate compounds, PHMB demonstrated the highest inhibitory activity against both bacterial strains (Table 2), preventing growth at concentrations of 0.00649 mg/mL (0.000625% *v*/*v*) and 0.0519 mg/mL (0.005% *v*/*v*) for *S. aureus* and *P. aeruginosa*, respectively. Curcumin solubilized in Polysorbate 40 showed the next highest inhibitory activity against *S. aureus* at 0.125 mg/mL (0.0125% *w*/*v*) and *P. aeruginosa* at 1 mg/mL (0.1% *w*/*v*). However, when curcumin is solubilized in ethanol, a higher concentration of 2 mg/mL (0.2% *w*/*v*) is required for the inhibition of bacterial growth of both *S. aureus* and *P. aeruginosa*. This was much greater than ethanol alone which had a MIC of 49.3125 mg/mL (6.25% *v*/*v*) for both strains, suggesting a major contribution of curcumin to bacteria growth inhibition. Retinol solubilized in ethanol has a MIC of 50 mg/mL (5% *w*/*v*) against both pathogens, slightly lower than that of ethanol alone. TPGS demonstrated inhibition of *S. aureus* and *P. aeruginosa* growth at 200 mg/mL (20% *v*/*v*), while Polysorbate 40 showed no inhibitory activity at >270.75 mg/mL (25% *w*/*v*). Collectively, PHMB was identified as the most effective antibacterial compound of those investigated.

Due to the low saturation solubility of retinol and curcumin in their respective vehicles, it was impossible to obtain a high enough concentration to observe a minimal bactericidal concentration (MBC). MBC values were also unattainable for both TPGS and Polysorbate 40 due to high viscosity preventing precise dosing. Only in the case of PHMB was it efficient enough at low concentrations to determine MBC values of 0.03125 mg/mL (0.003125% *w*/*v*) and 0.125 mg/mL (0.0125% *w*/*v*) for *S. aureus* and *P. aeruginosa,* respectively.

### 3.2. Dynamics of PHMB Antimicrobial Activity

**Figure 3:**
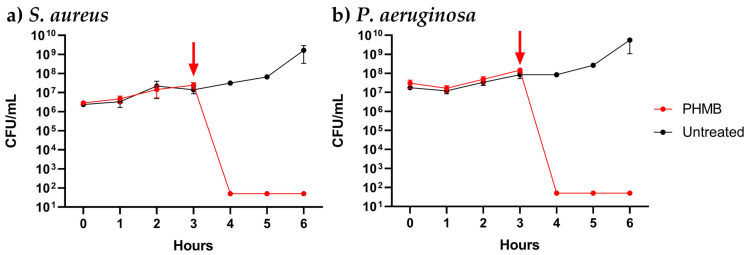
Bacterial growth kinetics of (**a**) *S. aureus* (NCTC12493) and (**b**) *P. aeruginosa* (NCTC12903) grown in Luria–Bertani (LB) following the dosing of the antiseptic candidate, PHMB (0.1%) at 3 h (indicated by the red arrow). The experiment was performed on three separate occasions for biological and technical triplicate.

Antibacterial compounds can interact with bacteria in a variety of ways; these include limiting the ability of the organism to replicate, known as bacteriostatic, or causing bacterial death or bactericidal activity [39]. In treating chronic wounds, the elimination of pathogenic bacteria is highly desired due to the reduced likelihood of reinfection [40]. As PHMB exhibited the most effective antibacterial activity against both *S. aureus* and *P. aeruginosa*, the dynamics of the bacterial killing were investigated. Viable bacterial counts were determined one hour following the addition of 0.1% PHMB to *P. aeruginosa* and *S. aureus* in liquid culture at the mid-log phase. The addition of PHMB resulted in the complete elimination of both bacteria after one hour (Figure 3). This confirms PHMB has a bactericidal effect upon both pathogens in their planktonic form, while also suggesting bacterial killing occurs rapidly. 

### 3.3. Biofilm Removal

Biofilm formation is frequently associated with infected chronic wounds, and therefore the ability of the selected compounds to prevent the formation of and allow the clearance of mature biofilms was investigated. Following the determination of the MICs and MBCs determined, PHMB, TPGS, TPGS-M, and ethanol were selected for further investigation of their anti-biofilm properties. Retinol was not considered further due to its high cost and high MIC, and curcumin was not considered further due to poor stability, resulting in reductions in antibacterial activity over time (Table S1).

**Figure 4:**
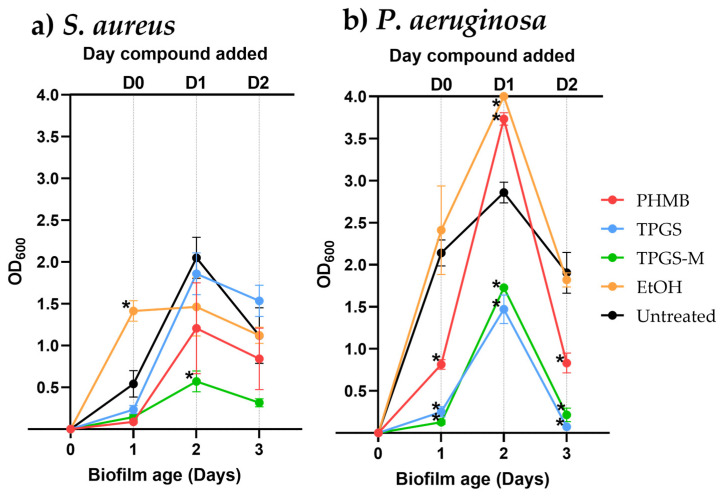
Biofilm growth kinetics following dosing of candidate compounds at MIC concentrations; PHMB, TPGS, TPGS-M, and Ethanol (EtOH), against (**a**) *S. aureus* (NCTC12493) grown in DMEM and (**b**) *P. aeruginosa* (NCTC12903) grown in M9 minimal media + 1% glucose. The results were determined by following the protocol detailed in Merritt et al., 2005 [37]. The experiment was performed 3 times for biological and technical triplicate Significance was determined by One way ANOVA and Dunnett’s test of multiple comparisons versus untreated * = *p* ≤ 0.05.

Without the addition of candidate compounds, biofilm production for both *S. aureus* and *P. aeruginosa* was observed to peak on day two before declining on day three (Figure 4), with this period used to represent a snapshot of in vitro biofilm dynamics. To investigate the ability to prevent biofilm formation, candidate compounds were administered on the day of biofilm seeded at D0. TPGS, TPGS-M, and PHMB individually resulted in significant reductions in *P. aeruginosa* biofilm formation (*p* ≤ 0.05) while also causing slightly decreased *S. aureus* biofilm formation at 24 h. However, administration of ethanol at D0 resulted in a marginal increase and significant increase in *P. aeruginosa* and *S. aureus* biofilm production at the point of analysis, respectively.

The disruption of developing biofilms was then investigated through the addition of compounds to biofilms at D1. All investigated compounds resulted in decreased *S. aureus* biofilm formation after 24 h, with TPGS-M having the most significant effect. In contrast, the addition of PHMB and ethanol resulted in significantly greater *P. aeruginosa* biofilm formation, while TPGS and TPGS-M resulted in significantly reduced formation after 24 h. To determine the effectiveness of the compounds against mature biofilms, they were added on day 2, D2. Unexpectedly, administration of TPGS resulted in significantly reduced *P. aeruginosa* biofilm formation after 24 h but increased that of *S. aureus*. Meanwhile, ethanol addition resulted in a comparable formation of biofilm for both bacteria. TPGS and TPGS-M also decreased both *S. aureus* and *P. aeruginosa* biofilms after quantification, with the latter being significant (*p* ≤ 0.05). Collectively, TPGS-M appeared to be the most effective at reducing biofilm formation for both bacteria, while TPGS was most effective against *P. aeruginosa* but was less so against *S. aureus*.

### 3.4. Compounds in Combination

The elimination of both bacteria colonizing the chronic wound area and existing biofilm structures is highly desirable in the treatment of infected chronic wounds. In this study, PHMB was observed to have highly effective antibacterial activity against both *S. aureus* and *P. aeruginosa*, yet a variable impact on *P. aeruginosa* biofilm removal. Therefore, in an effort to improve effectiveness against biofilms, PHMB was combined in formulation with TPGS, which was observed to effectively prevent the formation of and clear mature *P. aeruginosa* biofilms but lacked substantial antimicrobial activity against both pathogens.

When comparing the individual compound’s effectiveness to the combination, only TPGS’s activity was evaluated as we are unable to determine the stability and hence the presence of micelles in TPGS-M within a formulation. Hence utilizing TPGS establishes the baseline effectiveness of the combined formulation.

**Figure 5:**
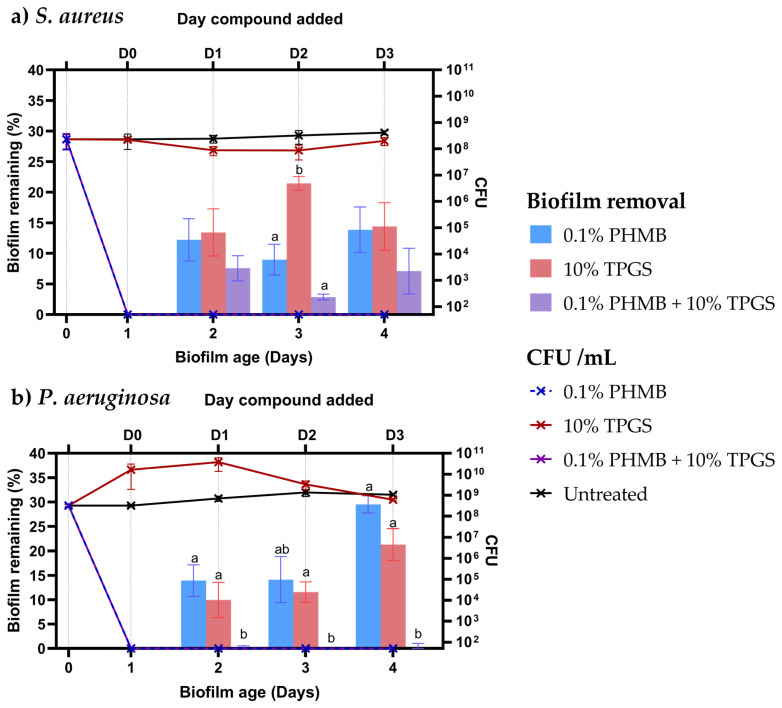
Bacterial growth kinetics of (**a**) *S. aureus* (NCTC12493) grown in DMEM and (**b**) *P. aeruginosa* (NCTC12903) grown in M9 minimal media + 1% glucose. The results for biofilm growth were determined by following the protocol detailed in Merritt et al., 2005 [37]. Experiments were performed on three occasions for biological and technical triplicate. Significance determined by One way ANOVA and Tukey’s multiple comparisons test, differing letters = *p* ≤ 0.05.

Application of the PHMB and the PHMB/TPGS formulation at all time points assayed (D0-D3) resulted in the complete elimination of both pathogens after 24 h of incubation (Figure 5). However, TPGS alone had little effect on *S. aureus* viability and resulted in increased *P. aeruginosa* colony counts on D1–D3 within the wells after 24 h. Administration of PHMB, TPGS, and PHMB/TPGS at the point of biofilm seeding (D0) resulted in the prevention of biofilm formation for both *S. aureus* and *P. aeruginosa* after 24 h (Figure 5). The application of each of the compounds/formulation to a maturing biofilm on D1 resulted in a reduction of over 85% of the *S. aureus* and *P. aeruginosa* biofilm as compared to the untreated control. However, this reduction was greater within biofilms treated with PHMB/TPGS formulation compared to each compound alone, with near-complete removal of *P. aeruginosa* biofilm 24 h later. Similarly, the addition of the PHMB/TPGS formulation to biofilms on D2 and D3 saw a greater impact on biofilm removal compared to PHMB and TPGS alone, with complete *P. aeruginosa* biofilm removal again occurring. Together, the PHMB/TPGS formulation allowed the effective elimination of both *S. aureus* and *P. aeruginosa* while also enhancing biofilm clearance compared to PHMB and TPGS alone.

## 4. Discussion

Our work here highlights the potential efficacy of a combinatory approach for the treatment of bacterial biofilms commonly associated with chronic wounds. We show that of the non-antibiotic compounds investigated, PHMB had the strongest inhibitory effect, with its presence at regulatory approved levels of 0.1% resulting in rapid bactericidal activity against both *S. aureus* and *P. aeruginosa* [41]. Furthermore, while not displaying any potent antibacterial activity, TPGS was shown to be the most effective candidate compound at preventing and removing *S. aureus* and *P. aeruginosa* biofilm formation, particularly when in a micellular form. We also successfully showed that a combination of PHMB and TPGS synergistically improved effectiveness against bacterial biofilms compared to the compounds administered alone. Novel management strategies are required for the treatment of bacterial infections within chronic wounds due to the emergence of multidrug-resistant bacteria. The work presented here highlights the utility of combining non-antibiotic compounds with multiple beneficial properties in formulation to enhance antibacterial and antibiofilm activity, which may potentially lead to the development of future treatment strategies.

Non-antibiotic compounds have demonstrated growing promise as treatments for chronic wound healing due to their antimicrobial, anti-inflammatory, or healing-promoting properties while also avoiding contributing to the development of AMR [31]. However, further investigation of the specific properties and interactions with pathogens is required to inform clinical applications. Here, the antimicrobial and antibiofilm properties of six compounds, retinol, curcumin, PHMB, TPGS, polysorbate 40, and ethanol, were characterized through the determination of MIC and CV biofilm clearance.

Retinoids, including retinol, have found use in a number of topical formulations aimed at controlling bacterial colonization of the skin and preventing infection [30]. Retinol has previously demonstrated no ability to inhibit the growth of MRSA (20 clinical strains) and *P. aeruginosa* (1 reference strain) in concentrations ≤0.128 mg/mL [30]. In comparison, we found retinol did exhibit antibacterial activity against both *S. aureus* and *P. aeruginosa* but at much higher concentrations of 50 mg/mL. While further work is required to determine the mechanism of action of retinol antibacterial activity, previous studies centered on the synthetic retinoic acid compounds CD437 and CD1530 identified an effective ability to disrupt the lipid biolayer of MRSA, leading to bacterial cell death [42], potentially suggesting a similar effect following retinol exposure. However, CD437 has also been observed to have no antibacterial activity against Gram-negative bacteria [43], unlike what was observed within this study against the Gram-negative *P. aeruginosa*, indicating retinol may have other, currently unknown effects upon bacteria. Curcumin is a bioactive compound derived from turmeric and has been observed to have effective anti-inflammatory and antimicrobial properties. Inhibition of both MRSA and *P. aeruginosa* has been observed at concentrations of 2–5 mg/mL and 0.0625–5 mg/mL, respectively [33]. Here, we show comparable curcumin MIC levels of 0.125–2 mg/mL against *S. aureus* and 1–2 mg/mL *P. aeruginosa*. Curcumin has been demonstrated to have broad-spectrum activity against both Gram-positive and negative bacteria, targeting a range of bacterial components, including the cell wall, cell membrane, and bacterial DNA, and interfering with the quorum sensing (QS) system [44]. This targeting of multiple bacterial characteristics is highly beneficial as it may reduce the ability of bacteria to develop resistance to curcumin [45]. However, the low solubility and instability of curcumin in an aqueous solution prove challenging, given the current in vitro analysis methods. PHMB has been demonstrated to have highly effective inhibitory activity against chronic wound-associated bacteria *S. aureus* and *P. aeruginosa*. Previously, PHMB was observed to have effective antibacterial activity against *S. aureus*, with an MIC of 0.001 mg/mL against three *S. aureus* strains, as well as almost complete elimination of *S. aureus* within HaCaT keratinocytes at concentrations of 0.004 mg/mL [23]. PHMB has also been demonstrated an MIC of 0.0156 mg/mL against four *P. aeruginosa* strains [46] and the capability to strongly inhibit the growth and biofilm formation of *P. aeruginosa*, but can be further enhanced through combination with other compounds [47]. The MIC values generated in this study are the same magnitude reported in the literature, 0.00649 mg/mL and 0.0519 mg/mL against *S. aureus* and *P. aeruginosa,* respectively. Due to PHMB’s low MIC values, the MBC value were also measured, determined as 0.0324 mg/mL and 0.130 mg/mL against *S. aureus* and *P. aeruginosa,* respectively.

PHMB was found to have the lowest MIC, with further assays observing the complete killing of both *S. aureus* and *P. aeruginosa* within one hour. This potent antibacterial activity is predicted to occur through the entry of PHMB into both Gram-positive and Gram-negative bacteria, followed by the triggering of chromosome condensation within the bacteria, leading to cell elongation and cell death [19]. Interestingly, this effect was observed to be specific to bacteria upon the addition of PHMB to mammalian cells having no similar effect on chromosome condensation due to PHMB becoming trapped in the endosomes [19,48]. Furthermore, Allen et al., 2004 [49] show that PHMB binds to nucleic acids, precipitating out. This ‘protein aggregation’ occurs in the presence of media that contain tryptone and peptone, i.e., rich media [47], hence an additional reason why minimal medias are required to grow biofilms if PHMB is present. When isolating the source of flocculation, peptone water was mixed with PHMB to observe the precipitation. Following this, sodium chloride (NaCl), the other component of peptone water, aside from peptone, was mixed with PHMB, and the solution became similarly cloudy. Yanai et al., 2011 [50] show that NaCl inhibits the antiseptic effects of PHMB, whilst, in combination with poloxamer 407, it retains its antiseptic capabilities. The same cloudiness appeared when determining PHMB’s MBC value due to the same reaction between NaCl, present in nutrient agar, and PHMB. Hence, this is the reason why the standard nutrient agar was changed to MHA.

Following the establishment of the MICs of the candidate compounds, their individual ability to prevent and disrupt biofilm formation was assessed at MIC levels. Due to their cost and instability, retinol and curcumin were not considered further. For the bacterial strain *S. aureus*, the untreated biofilm growth peaked at D1 (Figure 4), followed by a decrease in the biofilm present. This sharp increase at the start could be due to the minimal media causing a bacterial stress response. Yet, if no additional stresses are presented, then it is possible that the biofilm prevents the bacteria from further growth and is hence broken down. Empty TPGS micelles (TPGS-M) showed the highest reduction in biofilm produced by *S. aureus*. In contrast, TPGS, which was simply diluted with water, despite being the same compound, is less effective at removing biofilms. This may be due to the even structure/distribution of TPGS micelles that can help its penetration into the extracellular structure in the biofilm. In the case of ethanol, the *S. aureus* biofilm increased when ethanol was added on the same day as inoculation (D0). It has been reported in the literature that ethanol can increase the bacterial stress response [51]. The results demonstrated that TPGS-M was the most potent biofilm disruptor of both pathogens. As of writing, this is the first report to suggest that TPGS alone can disrupt bacterial biofilms [52]. Previously, TPGS encapsulation has been demonstrated to facilitate the deposition and penetration of fluorescent nanoparticles into biofilms. The enzymatic cleavage of TPGS by esterases into lipophilic vitamin E and hydrophilic PEG chains was seen to then release the nanoparticle into the biofilm [53]. However, this cleavage of TPGS may provide insight into a potential mechanism of action for TPGS biofilm prevention and removal, as vitamin E has been observed to significantly reduce the formation of biofilm by *S. aureus* and *P. putida*, as its release upon cleavage may induce a pharmacological effect [54]. Moreover, many antibiofilm compounds interfere with the QS system, in which complex physiological processes, including biofilm formation, are coordinated through the release of chemical signaling compounds named autoinducers by members of the microbial community [52,55]. Vitamin E, which can be generated from the cleavage of TPGS, has previously been observed to share structural similarities to the *P. aeruginosa* master QS regulator PqsR. Biofilm formation and virulence factor expression by *P. aeruginosa* was observed to be significantly reduced in the presence of Vitamin E, implying an impedance of the QS network [56].

Interestingly, the application of PHMB at MIC to *P. aeruginosa* biofilms on D1 resulted in significantly increased biofilm production after 24 h (Figure 4), yet when applied at higher concentrations does demonstrate antibiofilm properties (Figure 5). This contrasts with other studies in the literature which has investigated the antibiofilm properties of PHMB, which have collectively highlighted PHMB as an effective remover of biofilms [23,47]. Yet despite several studies, the mechanism of PHMB biofilm removal remains poorly understood, but the potential interaction of PHMB with the DNA-rich EPS may be a possible mechanism of action [23,47]. As mentioned previously, the addition of either PHMB or ethanol at lower concentrations may trigger a bacterial stress response for *P. aeruginosa,* and the same applies in the case of ethanol, which caused increased *S. aureus* biofilm formation.

Specifically, PHMB, which demonstrated rapid and effective antibacterial activity when combined with the biofilm-disrupting TPGS, results in a formulation with highly effective antibacterial and antibiofilm activity (Figure 5). These results highlight that PHMB, even at low concentrations, is a highly efficacious antibacterial and TPGS an effective biofilm remover [53]. However, neither compound alone is equally effective against both. The combined formulation would act to remove both the biofilm and bacteria, with TPGS disrupting the bacteria’s ‘protection’ and PHMB killing the bacteria.

Future work within this area should also factor in the ability to potentially enhance wound healing with retinol and curcumin to support the healing process [32,57,58]. Evaluating the formulations on in vitro and ex vivo models, especially on infected wound models, can help assess wound healing capabilities as well as antibacterial and antibiofilm properties, hence improving the overall understanding of this formulation together [34,59]. Since chronically infected wounds rarely harbor a single bacterial species, the next logical step would be to conduct the same experiments in vitro with coexisting bacteria in biofilms [60].

## Figures and Tables

**Table 1:** Individual compound properties and stock concentrations in the vehicle/solvent.

Compound	Molecular Weight	Purity/ Concentration	Solvent
Retinol	286.45	100 mg/mL; 10%	Ethanol
Curcumin	368.38	4 mg/mL; 0.4%	Ethanol
Curcumin	368.38	4 mg/mL; 0.4%	Polysorbate 40
Polyhexamethylene biguanide (PHMB)	- ^1^	208 mg/mL; 20%	water
D-α-Tocopherol polyethylene glycol 1000 succinate (TPGS) ^3^	- ^1^	200 mg/mL; 20%	Milli-Q water
D-α-Tocopherol polyethylene glycol 1000 succinate micelles (TPGS-M) ^3^	- ^1^	200 mg/mL; 20%	Milli-Q water
Polysorbate 40	1277 ^2^	100%	-
Ethanol	46.07	100% (>99.7%)	-

^1^ Polymer. ^2^ Estimated (Sigma Aldrich). ^3^ This has two forms, TPGS mixing with water and TPGS water micelles (TPGS-M).

**Table 2:** Minimum inhibitory concentrations of candidate antiseptic compounds against *S. aureus* (NCTC12493) and *P. aeruginosa* (NCTC12903) were determined by the broth microdilution method. Experiments were performed on two occasions, each in technical triplicate.

Compound (Concentration before Inoculation)	Vehicle	Bacterial Strain	
		*S. aureus*	*P. aeruginosa*
		MIC (mg/mL)	
Retinol	Ethanol	50	50
Curcumin	Ethanol	2	2
Curcumin	Polysorbate 40	0.125	1
PHMB	Water	0.00649	0.0519
TPGS	Water	200	200
Ethanol	-	49.3125	49.3125
Polysorbate 40	-	>270.75	>270.75

## Data Availability

The data presented in this study are available from the corresponding author upon reasonable request.

## Supplementary Materials

The following supporting information can be downloaded at: https://www.mdpi.com/article/10.3390/pharmaceutics15061633/s1, Table S1: Minimum inhibitory concentrations of curcumin at various formulation ages against *S. aureus* (NCTC12493) and *P. aeruginosa* (NCTC12903).
