# Supplementary material for: Non-Antibiotic Compounds Synergistically Kill Chronic Wound-Associated Bacteria and Disrupt Their Biofilms
Source: Pharmaceutics. 2023 May 31;15(6):1633. doi: 10.3390/pharmaceutics15061633 (PMC10302581; doi:10.3390/pharmaceutics15061633)
Supplement: Supplementary file 1 [file pharmaceutics-15-01633-s001.zip › pharmaceutics-2393971-supplementary.pdf]

pharmaceutics

Supplementary Materials: Non-Antibiotic Compounds Synergistically Kill Chronic Wound-Associated Bacteria and Disrupt Their Biofilms

Lucy Coleman, James R. G. Adams, Will Buchanan, Tao Chen, Roberto M. La Ragione and Lian X. Liu

Table S1. Minimum inhibitory concentrations of curcumin at various formulation ages against *S. aureus* (NCTC12493) and *P. aeruginosa* (NCTC12903).

Compound (concentration before inoculation)	Vehicle	Age	Bacterial strain	
			S. aureus	*P. aeruginosa*
			MIC (mg/mL)	
Curcumin	Ethanol	Fresh	2	2
Curcumin	Ethanol	9 days old	1	0.25
Curcumin	Ethanol	18 days old	0.5	1
Curcumin	Polysorbate 40	Fresh	0.125	1

Curcumin has a very low solubility in water; hence, ethanol was used as the vehicle instead (as was done with retinol). Following the solubilisation of the retinol and curcumin in ethanol, the broth microdilution assays were conducted to calculate the MICs. The experiments were repeated with the same compound sample as previously used. However, in the case of curcumin, it was observed that the MICs had each changed, more obviously in the case of *P. aeruginosa*. The microdilution assay was performed using different ‘ages’ of curcumin in ethanol. The overall trend that was observed was the older the sample, the lower the MIC. We hypothesis a reaction occurring between ethanol and curcumin, that deactivates the anti-bacterial functionality, degrading the efficacy over time. An alternative solubilising vehicle was tested, Polysorbate 40. Comparing the ‘fresh’ samples with the ethanol and polysorbate 40, the polysorbate 40 maintains the curcumins anti-bacterial property better than ethanol, shown by the lower MIC.

Due to this degradation issue, curcumin was not tested further, due to the prolonged experiment periods for biofilms and the subsequent crystal violet assay tests.
